# N-Acetylgalactosaminyltransferase-14 as a potential biomarker for breast cancer by immunohistochemistry

**DOI:** 10.1186/1471-2407-10-123

**Published:** 2010-04-01

**Authors:** Chen Wu, Xiaodan Guo, Weina Wang, Yun Wang, Yaojun Shan, Bo Zhang, Wenqian Song, Sisi Ma, Jianfeng Ge, Hao Deng, Mingsheng Zhu

**Affiliations:** 1 College of Life Sciences, Hebei University, Baoding, Hebei, 071002, PR China; 2 No. 3 Hospital, Baoding, Hebei, 071002, PR China; 3 College of Pharmaceutical Sciences, Hebei University, Baoding, Hebei, 071002, PR China

## Abstract

**Background:**

The post-translational modification of proteins, including glycosylation, differs between normal and tumor cells. The UDP-N-acetyl-D-galactosamine polypeptide N-acetylgalactosaminyltransferases (GalNAc-Tases) family of enzymes regulates the initial steps of mucin O-glycosylation and is responsible for the altered glycosylation state observed in cancer cells. Recently it was found that GalNAc-T14 mRNA is heterogeneously expressed in breast carcinomas compared to normal tissue, however the expression profile of GalNAc-T14 protein in breast carcinomas compared to normal tissue is still unknown. In this study, we assessed the expression profile of GalNAc-T14 protein in malignant and non-malignant breast tissues by immunohistochemistry to evaluate whether GalNAc-T14 might be a potential biomarker for breast cancer.

**Methods:**

In formalin-fixed tissues, the expression level of GalNAc-T14 protein was evaluated by immunohistochemistry assay in breast tissues. Expression profiles were assessed in normal tissues, benign fibroadenomas and several types of carcinomas.

**Results:**

Our results showed that GalNAc-T14 was heterogeneously expressed in breast carcinomas compared to non-malignant tissue. GalNAc-T14 expression was observed in 47/56 (83.9%) carcinoma samples, 7/48 (14.6%) non-malignant breast tissue samples. GalNAc-T14 expression level was associated with histological grade. For this enzyme a significant association with invasive ductal type, mucinous adenocarcinoma and ductal carcinoma in situ (DCIS) type was found.

**Conclusion:**

Our results provide evidence that GalNAc-T14 may be a potential biomarker for breast cancer by immunohistochemistry. GalNAc-T14 expression level was associated with histological grade. GalNAc-T14 expression can provide new insights about breast cancer glycobiology.

## Background

Polypeptide N-acetylgalactosaminyltransferase 14 (GalNAc-T14, EC 2.4.1.41) belongs to a large subfamily of glycosyltransferases residing in the Golgi apparatus. *N*-acetylgalactosaminyltransferases (GalNAc-Tases) catalyse the first step in the *O*-glycosylation of mammalian proteins by transferring *N*-acetyl-D-galactosamine (GalNAc) to peptide substrates. Fifteen distinct members of the mammalian GalNAc-Tases family have been identified and characterized [1-13]. Some of them are widely distributed in various human tissues, but others show tissue-specific distribution. The difference in tissue distribution and substrate specificity toward peptides are related to the diverse functions of these different subtypes of GalNAc-Tases. Now it is known that the GalNAc-Tase family is closely related to invasion, metastasis and proliferation of many carcinomatous cells. It is widely known that the O-glycosylation alters in malignant transformation [14]. Tumor development is usually associated to alterations in cellsurface carbohydrates. Glycosylation changes arising as a consequence of malignant transformation influence cell growth as well as differentiation, adhesiveness and immunogenicity of cancer cells [15]. Tumor-associated carbohydrate antigens are produced due to the deregulation of glycosyltransferases, resulting in changes in enzyme activity and specificity for specific substrates.

Altered expression of GalNAc-Tases could be one of the mechanisms that explain the changes in mucin *O*-glycosylation during malignant transformation [16]. Mucin glycoproteins (mucins) expressed on carcinoma cells representing a metastatic phenotype are qualitatively and quantitatively different from those of the nonmetastatic phenotype [17-19]. Alterations in the glycosylation of carcinoma-associated mucins may modulate the biological behavior of carcinoma cells and allow the cells to disseminate, invade and survive at distant organ sites [20]. These alterations include aberrant glycosylation, and incomplete glycosylation. The carbohydrate antigens on the cell surface are useful for monitoring tumor status in subjects with malignant diseases [21-24].

Variations of the GalNAc-Tases expression pattern have been described in several cell carcinomas. Akita *et al *suggested that GalNAc-T3 can serve as a new marker of non-small cell lung cancers with specificity for histology and prognosis [25]. Decreased expression of GalNAc-T1 and increased expression of GalNAc-T2 and GalNAc-T3 have been reported in oral squamous cell carcinoma when compared to the expression pattern in normal oral mucosa [26]. Higher expression of GalNAc-T1, GalNAc-T2 and GalNAc-T3 has also been described in colorectal carcinoma when compared with the normal colonic epithelium [27]. Different levels of expression of GalNAc-T3 were detected in patients with colorectal [12], lung [28], pancreatic [29], gastric [30], gallbladder [31], prostate [32] and extrahepatic bile duct carcinomas [33], and it was identified as an independent factor of prognosis. GalNAc-T6, which exhibits a high sequence homology to GalNAc-T3, has been recently described to be expressed in most ductal breast carcinomas but not in normal breast epithelium. This association is significant in the T1 tumor stage [34]. GalNAc-T3 and -T6 were also described in association with breast malignant cell lines [35]. GalNAc-T14 was first reported in 2003 by Wang *et al*, who cloned its cDNA, designated it as GalNAc-T14 and confirmed that it is a new member of the GalNAc-Tase family. Quantitative real-time polymerase chain reaction (PCR) analysis revealed that the GalNAc-T14 transcript was highly expressed in kidney tissue, which suggests that GalNAc-T14 might be involved in *O*-glycosylation in the kidney [10]. Wagner *et al *found that mRNA expression of the peptidyl O-glycosyltransferase GalNAc-T14 correlated with Apo2L/TRAIL sensitivity in pancreatic carcinoma, non-small-cell lung carcinoma and melanoma cell lines, and up to 30% of samples from various human malignancies including breast cancer, ovarian cancer, lung cancer and skin cancer showed GalNAc-T14 overexpression using real-time PCR assays. RNA interference of GalNAc-T14 reduced cellular Apo2L/TRAIL sensitivity, whereas overexpression increased responsiveness. These results uncover a new link between death-receptor O-glycosylation and apoptotic signaling, providing potential predictive biomarkers for Apo2L/TRAIL-based cancer therapy [36]. We have recently described the mRNA expression of GalNAc-T14 in human breast cancer cell lines and breast tumors. Immunohistochemical studies have shown an altered expression of some GalNAc-Tases in malignant transformation. In the present study we characterized the expression of GalNAc-T14 in non-malignant and malignant breast tissues using immunohistochemical assay. We demonstrated that GalNAc-T14 was expressed in most breast carcinoma and there were changes in its expression level during breast carcinogenesis. Expression of GalNAc-T14 was found to be associated with clinicopathological characteristics of breast carcinoma, therefore, GalNAc-T14 could be a specific biomarker for breast cancer.

## Methods

### Collection of tissue samples and histological classification

The study was performed using surgical specimens of breast carcinomas and breast adenosis from patients operated at No.3 Hospital (Baoding, China). The hospital institutional ethical review committee approved this study protocol, and all patients provided written informed consent. Analysis of the expression of GalNAc-T14 was performed in 104 breast tissue samples constituted by normal tissues (16), adenosis (32), invasive ductal carcinomas (43), lobular carcinomas (4), ductal carcinoma in situ (DCIS, 5) and mucinous adenocarcinoma (4) fixed in 10% neutral formalin and embedded in paraffin. Characteristics of breast cancer patients and tumors are listed in Table 1. These normal tissues which were more than 5 cm distant from carcinoma were from the same invasive ductal carcinomas patients. Serial sections were cut and used for conventional histopathologic diagnosis. Carcinomas were classified according to WHO (2003). Age, presence of lymphatic invasion, nodal metastasis and tumor localization were also recorded in every case. The pathologic staging was achieved using the WHO PTNM classification for breast carcinoma.

**Table 1:** Characteristics of patients and tumors

	Median (range)	n (%)
**Age in years**	51.8 (30-74)	
Histological type		
Invasive ductal		43 (76.80)
Invasive lobular		4 (7.15)
DCIS		5 (8.90)
Mucinous Adenocarcinoma		4 (7.15)
**Nodal status**		
Negative		32
Positive		24
**Histological grade**		
Grade 1		7
Grade 2		19
Grade 3		17
**Estrogen receptors**		
Negative		27 (48.2)
Positive		29 (51.8)
**Progesterone receptors**		
Negative		37 (66.1)
Positive		19 (33.9)
**ErbB2**		
Negative		30 (53.5)
Positive		26 (46.5)
**P53**		
Negative		6 (10.8)
Positive		50 (89.2)

### Immunohistochemistry

Immunohistochemical staining of GalNAc-T14 was performed in 88 randomly chosen patients who had undergone surgical treatment for a breast disease at No.3 Hospital (Baoding, China) between 2008 and 2009 (56 patients with histopathological diagnosis of breast cancer and 32 patients with non-malignant diseases). Elivision™ plus two-step system was used to detect GalNAc-T14 expression. Paraffin-embedded tissue was freshly cut (4 μm). The sections were dewaxed with xylene, and gradually hydrated in a decreasing ethanol series ending in distilled water. Endogenous peroxidase activity was quenched using 0.3% hydrogen peroxide in methanol for 30 min and then washed in phosphate buffered saline (PBS). Antigen retrieval was achieved using a pressure boiler heating in retrieval solution, pH 6, at 125°C for 4 min, followed by a 20 min cooldown period at room temperature. Sections were incubated with normal goat serum diluted 1:5 in PBS containing 10% of BSA for 20 min. After that, sections were incubated overnight at 4°C with the rabbit polyclonal antibody against the synthetic peptide derived from residues 50-105 of human GalNAc-T14 (Sigma) at the dilution of 1:15. After washing with PBS, staining was performed by the Elivision™ plus two-step system (Maixin Bio, China). Reactions were revealed with 3,3'-diaminobenzidine (DAB, Sigma). Slides were then counterstained with hematoxylin, washed, dehydrated with alcohol and xylene and mounted with coverslips. Between each step, sections were washed in PBS. For every assay, omission of the primary antibody and substitution by nonspecific immunoglobulin at the same concentration was used as negative controls. For ER, PR, and ErbB2 staining, sections were stained with the ER/PR/ErbB2/P53 immunohistochemistry test kit (Zhongshan Co., Beijing, China), which includes the mouse monoclonal antibodies against human ER, PR, ErbB2 and P53 proteins.

### Assessment of GalNAc-T14, ER, PR, ErbB2 and P53 expression

GalNAc-T14 expression is diffuse or granular staining in the cytoplasm. The immunostaining frequency for each tumor was scored as follows: 0, for negative samples or, 10%stained tumor tissue; 1, for samples stained between 10 and 39% of tumor tissue; 2, for samples stained between 40 and 79% of tumor tissue; and 3, for tumors with 80% of stained tumor tissue. Signal intensity was scored as strong (3, dark brown colour), moderate (2, medium brown colour), weak (1, light brown colour), and nul (0, no immunostaining). Total immunostaining score results from the multplication of both parameters. Samples were scored totally as follows: strong (+++, total immunostaining score = 6~9), moderate (++, total immunostaining scor = 3~4), weak (+, total immunostaining score = 1~2), and null (-, total immunostaining score = 0). Scores were established jointly by four observers under a multi-head microscope. Clinicopathological information was masked to the observers. ER, PR and P53 staining was scored in a minimum of 300 histologically identified neoplastic cells, showing nuclear reaction. Tumors with positive ER, PR or P53 nuclear staining in >10% of tumor cells were defined as ER, PR or P53 positive, respectively. For ErbB2 protein, immunostainings were scored as strong (2++ and 3+++), weak or negative (1+ and 0) according to the rate of labelled tumor cells and the membrane staining intensity.

### Statistical Analysis

Statistical analysis was performed using standard statistical software SPSS version 13.0 (Chicago, Illinois). Correlation between GalNAc-T14 expression and various clinicopathological factors were analyzed using Fisher's exact test. *P *< 0.05 was considered statistically significant.

## Results

**Figure 1:**
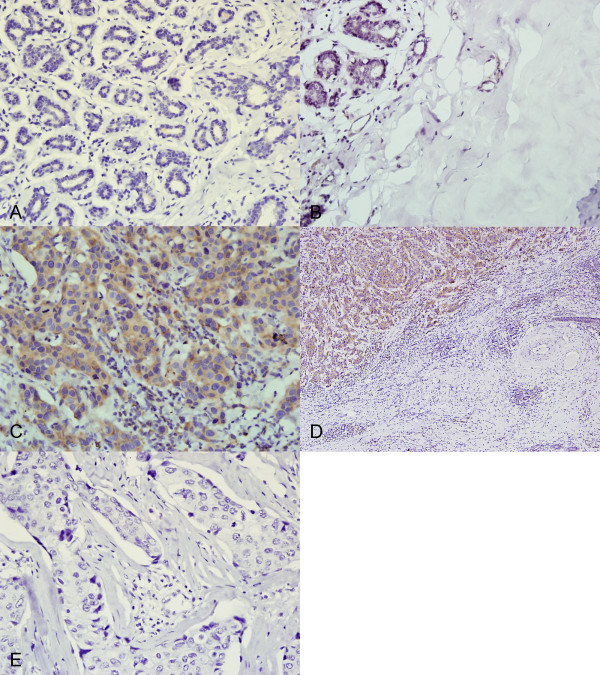
**Immunohistochemical evaluation of GalNAc-T14 in breast carcinomas and normal tissue**. A, Adenosis. B, Normal. C, Cancer. D, Cancer and surrounding normal tissue. E, Nonspecific immunoglobulin was used as a negative control. Magnification: A, B, C, E ×400; D ×100. (In order to show the immunostaining profile of carcinomas and normal tissue surrounding carcinomas in the same microscopic field, 100× magnification was used)

**Figure 2:**
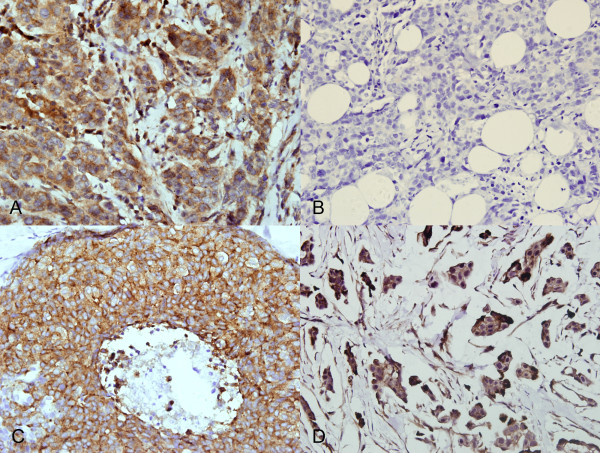
**GalNAc-T14 expression in different breast cancer type**. A, Invasive ductal carcinoma. B, Invasive lobular carcinoma. C, DCIS. D, Mucinous Adenocarcinoma. Magnification: ×400.

GalNAc-T14 expression was evaluated in 56 primary tumors from breast cancer patients (43 ductal carcinomas, 4 lobular carcinomas, 5 DCIS and 4 mucinous adenocarcinoma), 32 specimens from non-malignant breast diseases, and 16 normal breast tissues. The results of the immunohistochemical study of 104 breast tissues are summarized in Table 2. The majority of breast cancers were invasive ductal carcinoma. The breast carcinoma cells showed perinuclear of diffuse cytoplasmic immunostaining of GalNAc-T14 (Figure 1C-D). As illustrated in Table 2, there was a significant correlation between the expression of GalNAc-T14 and breast carcinomas. GalNAc-T14 was expressed more obviously frequently in cancers than in non-malignant tissues. GalNAc-T14 expression was observed in 47/56 (83.9%) carcinomas, whereas only 7/48 (14.6%) showed GalNAc-T14 expression in non-maglinant tissues (*P *= 0.000) (Table 2). GalNAc-T14 expression in different breast cancer types were displayed in Figure 2. Invasive ductal carcinoma was observed in 43/56 cancers, of which 37 (86.0%) were GalNAc-T14 positive (8/37 showed a strong immunostain, 11/37 immunostained moderately, and 18/37 immunostained weakly). Four invasive lobular carcinomas were evaluated and three (75%) were GalNAc-T14 negative, whereas one was immunostained weakly. The result is in agreement with GalNAc-T14 expression revealed by real-time quantitative PCR in several types of breast carcinomas tissues [36].

**Table 2:** Correlation of GalNAc-T14 expression with non-malignant tissues and carcinomas

Tissue type	Total number	Cytoplasm GalNAc-T14 expression *		P value
			
		Positive (%)	Negative (%)	
non-malignant	48	7 (14.6%)	41 (85.4%)	0.000
carcinomas	56	47 (83.9%)	9 (16.1%)	

* The positive cytoplasmic immunohistochemical reactivity of GalNAc-T14 in breast carcinomas includes strong, moderate and weak staining.

GalNAc-T14 expression was also evaluated in 5/5 (100%) adjacent ductal carcinoma in situ (DCIS) and 4/4 (100%) mucinous adenocarcinoma. Of the 5 DCIS, 4 (80%) were immunostained strongly and 1 (20%) was immunostained moderately. Three of four mucinous adenocarcinomas were immunostained strongly and one was immunostained weakly. In contrast to the positive expression frequently observed in breast cancers, GalNAc-T14 was rarely detected in non-malignant breast diseases. Only 7/48 (14.6%) of non-malignant tissues showed GalNAc-T14 expression, distributed as follows: 5/32 adenosis, 2/16 normal breast tissues. In order to detect the difference of GalNAc-T14 expression between normal tissues and benign tissues, 32 specimens from non-malignant breast diseases and 16 normal breast tissues that were from the cancer patients were analyzed. No difference was observed between them (Figure 1A-B). As showed in Figure 1D, GalNAc-T14 has a strong cytoplasmic immunostaining in carcinomas and no cytoplasmic immunostaining was observed in normal tissue surrounding carcinomas. No staining was observed with nonspecific immunoglobulin as a negative control (Figure 1E).

**Figure 3:**
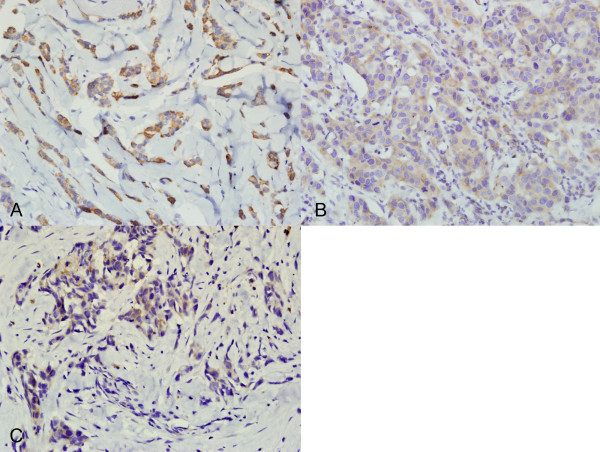
**The differential expression of GalNAc-T14 protein in breast carcinomas with different histological grades**. A, the cases with histological grade 1. B, the cases with histological grade 2. C, the cases with histological grade 3.

We observed a statistically significant association between GalNAc-T14 expression and tumor grading in invasive ductal carcinomas. High expression of GalNAc-T14 was found frequently in G1 tumors than G2 and G3 tumors. The relationship between GalNAc-T14 expression and clinicopathological factors is shown in Table 3. The cases with higher histological grade showed lower level of GalNAc-T14 expression (Figure 3). The expression level of GalNAc-T14 was high in 6/7 (85.7%) of G1 tumors, moderate in 9/19 (47.4%) of G2 tumors, and low (14/17, 82.4%) or null (2/17, 11.8%) in G3 tumors (p < 0.05). No correlation was found between GalNAc-T14 expression and ER, PR, ErbB2 protein levels. The relative expression level of GalNAc-T14 was not correlated with the expression of P53.

**Table 3:** Relationship between GalNAc-T14 expression and clinicopathological factors

Histopathological diagnosis	Grade	Sample number	GalNAc-T14 staining		
			
			Low	Moderate	Strong
Invasive ductal carcinoma	1	6			+++
	1	1		++	
	2	2			+++
	2	9		++	
	2	4	+		
	2	4	0		
	3	1		++	
	3	14	+		
	3	2	0		
Mucinous Adenocarcinoma		3			+++
		1	+		
Invasive lobular carcinoma		3	0		
		1	+		
DCIS		1		++	
		4			+++
Normal beast tissue		14	0		
		2	+		
Adenosis		27	0		
		4	+		
		1		++	

In immunocytochemical studies, GalNAc-T14 antibody was reactive with MCF-7 breast cancer cell lines (data not shown). The result was in agreement with GalNAc-T14 expression revealed by semi-quantitative RT-PCR in the cells lines (unpublished data).

## Discussion

Abnormal *O*-glycans expressed by cancer cells have functional importance in cell adhesion, invasion, and metastasis [15]. Alterations in mucin-type *O*-glycans has been associated with malignant transformation, resulting in the formation of less complex structures and leading to an increase of the simple short determinants. Protein *O*-glycosylation is deregulated in breast cancer cells, leading to the accumulation of simple mucin-type tumor-associated antigens [37]. The expression of GalNAc-T14 mRNA was analyzed in normal and malignant tissue from breast, skin, lung, pancreas, ovary, endometrium, bladder and lymphoid cancers. A subset of tumor samples, ranging from 10% in lobular breast cancer to 30% in lung cancer and diffuse large B-cell lymphoma, showed GalNAc-T14 mRNA overexpression [36]. Under thees circumstances, we hypothesize the expression of GalNAc-T14 may be a useful biomarker for breast cancer by immunohistochemistry.

It has been shown that several glycosyltransferases are useful tumor markers. Owing to the later discovery of this enzyme, GalNAc-T14 was rarely studied. Up to now, the expression of GalNAc-T14 protein in breast cancer has not been reported. In the present work we used a polyclonal antibody against this enzyme, to evaluate the potential role of GalNAc-T14 as a breast cancer biomarker. In the immunohistochemical study presented here, we described for the first time the expression of GalNAc-T14 protein in breast cancer. Our results showed that GalNAc-T14 is expressed in only 7/48 samples (14.6%) of non-malignant breast tissue, whereas it is expressed in most breast carcinomas (47/56, 83.9%). These results are in agreement with recent mRNA data that were obtained from non-malignant and malignant breast tissues using semi-quantitive RT-PCR in our laboratory (data not shown). It was found that the expression of GalNAc-T14 in breast invasive ductal carcinomas was associated with histological grading. Higher histological grading corresponded to lower expression level of GalNAc-T14.

Expression levels for GalNAc-T14 mRNA have been detected in human normal tissue and tumor tissue samples, including skin, pancreas, lung, breast, ovary, endometrium, bladder and lymph. Expression of the mRNA transcript which encodes the O-glycosylation initiating enzyme GalNAc-T14, was markedly higher in carcinoma tissue of lung, breast, ovary, endometrium, bladder versus normal tissue of those [36]. The members of the GalNAc-Tases family, GalNAc-T1, -T2, -T3, -T4 and -T6 were detected in a range of breast cell lines by immunocytochemistry with confocal scanning laser microscopy. The cells were chosen to represent a range of phenotypes from 'normal'/benign (HMT 3522), primary, non-metastatic breast cancer (BT 474), to aggressive, metastatic breast cancer (ZR75-1, T47D, MCF-7, DU 4,475). GalNAc-T1 and -T2 were detectable at low levels in all cell lines studied. GalNAc-T4, which has never been described in breast, was very weakly detectable in BT 474, MCF-7 and T47D. GalNAc-T3 and -T6 were weakly detectable or undetectable, respectively, in the cell line HMT 3522 derived from normal/benign breast epithelium, but were readily detectable in all malignant cell lines. Thus, a broader range of GalNAc-Tases were detectable in the malignant cell lines in comparison to the 'normal'/benign cells, where only the housekeeping GalNAc-T1 and -T2 were present. Expression of normally tightly restricted GalNAc-Tases may result in initiation of *O*-linked glycosylation at normally unoccupied potential glycosylation sites leading to altered glycoforms of proteins with changed biological activity which may contribute to the pathogenesis of cancer [35]. Different levels of GalNAc-T2 have also been detected in oral squamous cell carcinoma and colorectal carcinoma, and this has been associated with a poor prognosis. GalNAc-T14 exhibits a high amino acid sequence homology to GalNAc-T2, but *in vitro *studies have indicated that the most preferred glycosylation site within Muc5AC by GalNAc-T14 was different from the site preferred by GalNAc-T2. So the catalytic profiles of GalNAc-T14 may be different from that of GalNAc-T2. Our results suggest that GalNAc-T14 also may play a role in the biological characteristics of breast carcinoma cells, most probably through the variation in mucin *O*-glycosylation. We hypothesize that invasive ductal carcinoma with high histological grading would likely result in invasion and metastasis due to the downregulation of GalNAc-T14 expression. This occurence induces an incomplete elongation of *O*-glycan saccharide chains in mucins that can lead to the expression of shorter carbohydrate structures, such as the TF, sialyl-Tn, or Tn antigens. Tn antigen determinant (GalNAc-*O*-Ser/Thr: the innermost *O*-linked structure), which is usually masked by additional sugar residues in normal tissues, was characterized as one of the most specific human cancer-associated structures, and it was detected in approximately 90% of human carcinomas [38] A direct correlation has been shown between carcinoma aggressiveness and the density of expression of Tn in the tumor [39]. Thus, expression of the Tn determinant could be the result of glycosyltransferases deregulation via changes in enzyme activity and/or in substrate specificity [40]. The other possible reason is that both *O*-glycosylation and phosphorylation take place on the same catalytical sites on Ser/Thr residues. Therefore, *O*-glycosylation may competitively inhibit the Ser/Thr phosphorylation. Abnormal expression of GalNAc-T14 may result in initiation of *O*-linked glycosylation at normally unoccupied potential phosphorylation sites leading to altered proteins with changed biological activity which may contribute to the pathogenesis of cancer.

## Conclusions

In summary, our result is in line with previous studies showing that breast carcinoma tissue GalNAc-T14 mRNA was expressed heterogeneously in breast cancer. The abnormal expression of GalNAc-T14 in breast carcinomas may have induced changes in cellular functions including adhesion and invasion. Our results provide evidence that GalNAc-T14 may be a potential biomarker for breast cancer by immunohistochemistry. Moreover, the expression levels of GalNAc-T14 were associated with histological grade in breast invasive ductal carcinoma and tumour progression. For this enzyme a significant association with invasive ductal type, mucinous adenocarcinoma and DCIS type was found. Considering that abnormal *O*-glycosylation greatly contributes to the phenotype and biology of breast cancer cells, our results contribute to understanding the molecular mechanisms that underlie aberrant glycosylation in breast carcinogenesis and in breast carcinoma. To extend our observations, a follow-up study of a larger number of cases is necessary to determine the potential clinical value of this marker. Further studies are also required to determine the biological role of GalNAc-T14 in breast tumor development, growth, invasion and metastasis.

## Competing interests

The authors declare that they have no competing interests.

## Authors' contributions

CW designed the study, carried out immunohistochemical study, and drafted the manuscript. XDG, participated in study design, data acquisition, and drafting of the manuscript. WNW participated in the immunohistochemical studies. YW collected clinical specimens, recruited patients. YJS participated in the statistical analysis. BZ, WQS, SSM, JFG and HD participated in data acquisition and analysis. MSZ participated in the design of the study, supervised the laboratory work. All authors read and approved the final manuscript.

## Pre-publication history

The pre-publication history for this paper can be accessed here:

http://www.biomedcentral.com/1471-2407/10/123/prepub
